# The effects of mobile phone dependence on athletic performance and its mechanisms

**DOI:** 10.3389/fpsyg.2024.1391258

**Published:** 2024-05-16

**Authors:** Zhengyang Mei, Yuanzhuo Zhang, Qing Fan, Shulai Luo, Shi Luo

**Affiliations:** School of Physical Education, Southwest University, Chongqing, China

**Keywords:** athlete, mobile phone dependence, sports performance, neurobiological mechanism, theoretical model

## Abstract

Mobile phone dependence (also known as internet dependence, MPD), defined as a problematic behavior characterized by excessive use or intermittent craving to use a mobile phone, results in various social, behavioral, and affective problems in daily life. In sports, MPD is directly related to the physical and mental health and sports performance of athletes. The individual and environmental factors, neurobiological mechanisms and theoretical models of MPD affecting athletic performance were analyzed by reviewing previous studies, aiming to construct effective training and development protocols to prevent and control the occurrence of MPD in athletes. At present, athletic performance can be affected by MPD through individual factors and environmental factors. The neurobiological mechanisms between the two are based on the brain reward system and microwave radiation from mobile phones, with athletic performance being restricted by alterations in the corresponding brain regions. Relevant theoretical models mainly include the social cognitive model of self-regulation and the integrative model of self-control, which explain the interrelationship between MPD and athletic performance from the perspectives of athletes’ self-regulation and self-control, respectively. As an emerging phenomenon, the influence pathways and mechanisms by which MPD affects athletic performance need to be further investigated. A longitudinal perspective should be adopted to trace the dynamic impact relationship between the two, and developing relevant theoretical frameworks from an interdisciplinary research perspective should be valuable for providing theoretical support for coaches and sports administrators to formulate scientific training protocols and thus improve the mental health of athletes.

## 1 Introduction

According to the Global Digital Report, the number of unique mobile phone users sits at 5.61 billion at the start of 2024. The latest data from GSMA Intelligence reveals that 69.4% of the world’s total population now uses a mobile device. Meanwhile, the 53rd Statistical Reports on Internet Development in China released by the China Internet Network Information Center (CNNIC) shows that the number of internet users in China, as of December 2023, has reached 1.092 billion, with 3.8 and 14.7% of these users being under the age of 10 and between the ages of 10 and 19, respectively. The internet has nearly 200 million adolescent users, and the internet penetration rate among minors has almost reached saturation. With the increasing use of mobile phones, the associated problems and challenges have become more prominent. Additionally, there is a growing number of individuals who are addicted to, dependent on, or misusing mobile phones (Yu and Sussman, 2020). Mobile phone dependence (MPD) defined as a problematic behavior characterized by excessive use or intermittent craving to use a mobile phone, results in various social, behavioral, and affective problems in daily life (Billieux, 2012; Billieux et al., 2015). Moreover, some studies have adopted the synonymous concepts of MPD, including smartphone dependence (Park, 2019), smartphone addiction (Lopez-Fernandez, 2017), problematic mobile phone use (Harris et al., 2020), and mobile phone overuse (Kawyannejad et al., 2019).

Studies have demonstrated that the frequency and duration of mobile phone usage are important indicators for assessing MPD, and individuals who use mobile phones for more than 4 h per day can be considered potential mobile phone dependents (Song et al., 2023). At present, models of the influence mechanisms of internet addiction have been developed in the field of psychology, which indicate that internet addiction may lead to anxiety and depression, making it difficult to respond to life stress or negative events in a reasonable manner, and that psychological symptoms and pain can be worsened by such behavioral addictions. In the field of sports, mobile phones have significantly improved the lives and training of athletes, who regularly use mobile phones to manage roles and demands across multiple contexts, such as society, family and school (DesClouds and Durand-Bush, 2021). For young athletes, however, a range of physical and mental health problems can easily develop due to MPD, including emotional disturbance, poor sleep quality, difficulties in interpersonal relationship, reduced cognitive function, and attention allocation, which in turn can lead to impaired athletic performance (Encel et al., 2017; Gould et al., 2020; Jones et al., 2021; Ayyildiz and Besler, 2022; Gocer and Oniz, 2023). In recent years, MPD has become a highly significant research topic and has received increasing attention within the filed of sports psychology.

Therefore, this paper analyses and discusses the effects of MPD on athletic performance, the neurobiological mechanisms and relevant theoretical models of MPD affecting athletic performance, and on the basis of which research prospects are proposed, providing a theoretical reference and guidance for subsequent research in this field.

## 2 Relevant research on the effects of MPD on athletic performance

According to the holistic model of determinants of sports performance proposed by Bangsbo (2015), sports performance can be impacted by individuals’ psychological characteristics, physiological states, and external environmental conditions. Similarly, the effects of MPD on athletic performance can also be realized through interactions between individual and environmental factors. For instance, athletes may not be able to focus on training and lose the desire to pursue outstanding athletic performance due to interference with the attention allocation disrupted by MPD. Moreover, athletic performance can be restricted by MPD through weakening positive incentives in the sports environment, including an active training atmosphere, and friendly coach-athlete relationship (CAR). On this basis, the relevant research on the effects of MPD on athletic performance is divided into individual and environmental factors for discussion.

### 2.1 Individual factors

Athletes not only have to cope with the challenges and pressure of their peers, such as academic task, but also have to weather the additional demands of sports, and invest a great deal of time and energy in training and competition, thus they are subjected to various sources and forms of physical and psychological stress, including losing competitions, sports injuries, interpersonal relationships, further education pressure, future prospects, and personal life encounters (Fiedler et al., 2023). According to the general stress theory, problematic behaviors (e.g., MPD) are rooted mainly in negative emotions caused by stress or stressors, and the corresponding adaptive means will be adopted by athletes with problematic behaviors to alleviate pressure during stressful states (Agnew, 1992; Jun and Choi, 2015). Evidence has suggested that MPD tendencies may be more likely to develop in groups experiencing stress (Mehmood et al., 2021; Zhong et al., 2022). Athletes are susceptible to multiple stressors in life, study and training, which can lead to negative emotions such as anxiety and depression, and mobile phone usage provides an effective means for relieving stress. However, as most athletes have weak self-control, the psychological stress and negative emotions may be further aggravated by excessive use or dependence on mobile phones, which can adversely affect athletic performance (Elhai et al., 2017; Sahu et al., 2019; Ong et al., 2022), and even “choking” during competition. Ivarsson et al. (2017) have argued that adverse physiological reactions can be caused by the stress and anxiety experienced by athletes, including increased muscle tension, mental toughness and reduced neurocognitive and perceptual ability, with athletic performance being impaired. Furthermore, Hamlin et al. (2019) suggested that once the stress generated in academic and training environments exceeds athletes’ abilities to cope, athletes become more susceptible to decreased performance, increasing the risk of injury and illness. As a result, stressed athletes may view mobile phone usage as a kind of avoidant coping that allows them to temporarily escape from stressful situations, which in the long run may exacerbate MPD, rendering it unable to have adaptive coping effects, but further worsening psychological pressure and negative emotions, with athletic performance being constrained accordingly and difficult to improve.

Chronic problematic use of mobile phones may also consume the attentional resources and cognitive abilities of athletes (DesClouds and Durand-Bush, 2021; Ong et al., 2022), leading to mental fatigue and limiting athletic performance in training and competition (Fortes et al., 2022a; Alix-Fages et al., 2023). An 8-week randomized and experimental research with parallel groups showed that swimmers who used a smartphone for 30 min before training showed higher levels of mental fatigue (*p* = 0.01) and internal training load (*p* = 0.01), and their 100-m and 400-m freestyle performance gains were inhibited compared with the control group (Fortes et al., 2022b). According to the brain drain hypothesis proposed by Ward et al. (2017), using attentional resources for one cognitive process or task will reduce the resources available for other tasks. Simultaneously, since limited attentional resources are required to support other attentional control and cognitive processes, the attentional resources occupied by mobile phone usage will not be available for other tasks whose performance will suffer. Moreover, due to the mental fatigue and cognitive decline caused by prolonged exposure to smartphone apps, such athletes’ decision-making performance tend to be inhibited while engaging in tasks (Fortes et al., 2019). According to a randomized and experimental research with parallel groups on volleyball players, there was a statistically significant group × time interaction for both attack decision-making performance (*p* = 0.03) and passing decision-making performance (*p* = 0.02). Only athletes in the control group showed improvement in their attack decision-making performance (*p* = 0.02) and passing decision-making performance (*p* = 0.01) compared with athletes in the experimental group who used social media apps on smartphones before training (Fortes et al., 2021). These data corroborate the argument that sustained high cognitive demanding activity (e.g., MPD) led to mental fatigue and impaired decision-making performance in athletes (Smith et al., 2016; Gantois et al., 2020; Trecroci et al., 2020; Staiano et al., 2024). This may be due to the lack of self-regulation in such athletes, with the majority of limited attentional resources being focused on behavioral dependencies, resulting in fewer resources being allocated to athletic performance, reduced decision-making, and thus failure to achieve an optimal state of performance. In conclusion, for athletes with MPD, a large amount of attentional resources may be consumed by prolonged and high frequency mobile phone usage, reducing resource allocation to other cognitive activities, such as training and competition, leading to distraction, wandering and ultimately an inability to focus on current activities (Encel et al., 2017; David et al., 2018).

A body of research has indicated that athletic performance can be impaired by MPD through causing the sleep disturbance and irregular lifestyle (Thun et al., 2015; Romyn et al., 2016; Charest and Grandner, 2020; Gupta, 2023). Sleep is often considered as the key to achieving the optimal athletic performance, and sleep deprivation leads to a variety of physical and psychological consequences, including stress, anxiety, and decreased coping and recovery. Based on the blue light theory, the production of melatonin may be inhibited by the blue light emitted by mobile phones, thus increasing sleep latency and reducing sleep quality in athletes (Jahrami et al., 2022). Moreover, exposure to blue light also seems to activate the ventrolateral and dorsolateral areas of the prefrontal cortex, which interferes with sleep by increasing alertness and working memory (Bano et al., 2021). Investigating the relationship between sleep deprivation and muscle glycogen, Skein et al. (2011) suggested the storage of muscle glycogen before exercise will be reduced after sleep deprivation, because the sleep deprivation prevents the replenishment of muscle glycogen after exercise, damages the energy supply for muscle fibre repair and contraction, and subsequently leads to a decline in endurance athletic performance. Watson and Brickson (2018) assessed the relationships between sleep quality, training load and emotional state in female soccer players over a year, and reported that reduced sleep time was positively associated with fatigue (*β* = 0.15, *p* < 0.001), mood (*β* = 0.13, *p* < 0.001), and stress (*β* = 0.13, *p* < 0.001). An experimental control study investigating the effects of 24 h of sleep deprivation on youth soccer skills showed that athletes in the sleep deprivation condition exhibited higher levels of subjective sleepiness and distraction, with impaired athletic performance on seven soccer skills tests (Pallesen et al., 2017).

In summary, previous studies have examined individual factors categorized into multiple aspects, each of which is based on different intermediate variables to explore the effects of MPD on athletic performance. Prolonged use of electronic devices has been proven to be a behavior that requires high cognitive inhibition and sustained attention. When athletes use electronic devices, the cognitive performance will be impaired, including disrupt attention, concentration, memory, and executive function (Durand-Bush and DesClouds, 2018). It is well-documented that these cognitive deficits and increased cognitive demands for a prolonged period may impair decision-making performance and executive functions in athletes, which in turn may limit athletic performance (Van Cutsem et al., 2017; Fortes et al., 2019; Gantois et al., 2020). Therefore, the following aspects for expansion could be considered in future research: (1) Multiple individual factors, including mental toughness, personality disposition, coping style, attachment, and self-esteem, should be fully incorporated into research on MPD to examine the specific conditions under which MPD plays a restrictive role in athletic performance, facilitating a detailed exploration of the influence paths and corresponding intervention programs from different perspectives; (2) Based on the results of cross-sectional studies, longitudinal and long-term follow-up research should be conducted to examine the influence relation between the two in a more systematic and dynamic way; and (3) Targeted intervention experiments, including psychological intervention (Malinauskas and Malinauskiene, 2019), mindfulness training (Lan et al., 2018), educational intervention (Khoshgoftar et al., 2019) and web-based group intervention (Brouzos et al., 2024), could be designed and implemented to examine the withdrawal effects of different interventions on athletes with MPD.

### 2.2 Environmental factors

Research on the impacts of MPD on athletic performance through environmental factors is primarily based on the self-determination theory’s perspective on individual needs (Deci and Ryan, 2013). This theory classifies basic human psychological needs into three categories: relationship needs, autonomy needs and competence needs (Deci and Ryan, 2013). In essence, the communication between athletes and the outside world is a process of need fulfillment. According to the theory of compensatory internet use, most athletes are mentally immature, making them less capable of emotional regulation and socialization (Kardefelt-Winther, 2014). Consequently, they may find it challenging to meet their needs in real-world situations. Huang et al. (2022) found that social communications tend to be neglected by individuals with MPD, resulting in less time for offline communication with friends and family. This negatively affects the social–emotional support system, leading to alexithymia and increased loneliness. As a result, they may resort to inappropriate ways to meet social needs. Furthermore, individuals with MPD also tend to disregard their surroundings and develop a dependence on the virtual world accessed through mobile phones, with the execution of daily physical activities being affected. In sports, athletes with MPD may be influenced by the aforementioned behaviors, leading to formation of poor interpersonal relationships and incorrect interaction patterns, which are detrimental to the development of CAR and also hinder athletic performance. Evidence suggested that the interpersonal conflict within a team and poor team training atmosphere can easily develop through poor CAR, discouraging athletes from striving for excellence (Wachsmuth et al., 2018).

Moreover, burnout in athletes will correspondingly develop due to uncoordinated CAR, which will cause athletes to lose motivations to focus on training and competition (Isoard-Gautheur et al., 2016). In contrast, the 3Cs model proposed by Jowett and Cockerill (2003) suggested that the more satisfying the relationship between athletes and coaches is, the greater the quality of that relationship and the better athletic performance. According to the model of sports engagement, when establishing high-quality CAR, athletes will receive more informational, emotional, respectful, and instrumental support from coaches, which will help them meet sports needs, achieve a dynamic balance between them and the environment, and ultimately improve athletic performance. Therefore, athletes with MPD may not be able to build a harmonious CAR due to their own poor relationship building and communication skills, which in turn has a negative impact on athletic performance. For athletes with MPD, harmonious CAR is difficult to build and develop due to poor relationship building and poor communication skills, with a corresponding adverse impact on athletic performance.

The uses and gratifications theory posits that athletes use social media for a variety of needs, and mobile phones, as a “ritualized” medium, can be used to satisfy athletes’ needs to pass time, socialize and entertain, which are easy habits to develop (Katz et al., 1973). In the highly digital context, the convenience, entertainment and social functions offered by mobile phones may become a “psychological refuge” for athletes, allowing them to fully immerse themselves in social media interactions. However, when moving from the online world to the real world, such athletes may find it difficult to adjust their control and execution abilities, which can adversely affect athletic performance. A randomized and cross-over investigation of the inhibitory control performance (including accuracy and response time) showed that the mental fatigue caused by smartphone social media use resulted in higher response time than the control group at 10 min (*p* = 0.01) and 30 min (*p* = 0.01) after the resistance exercise session, suggesting that performing a high cognitive demanding activity (e.g., MPD) before training may trigger impaired inhibitory control performance (Lima-Junior et al., 2024). This evidence is consistent with findings from a randomized and experimental research with parallel groups, which found that the acute effects associated with 30 min of social media use before training interfered with the improvement of inhibitory control performance in athletes (Fortes et al., 2022b). The self-presentation model of social anxiety can be used to explain how social media affects athletic performance, stating that athletes attempt to present an idealized self-image to the public through online interaction, and that athletes will maintain their own self-image through frequent interaction when they are concerned about what others’ opinions are, or when the ideal impression is questioned (Schlenker and Leary, 1982). As a result, athletes easily fall into a state of distraction and anxiety, and have difficulty focusing on training and competition. This finding conforms with the cognitive resource allocation theory, which suggests that most psychological resources will be devoted to processing trivial and cumbersome information due to athletes’ frequent interactions with social media, giving rise to an under-allocation of localized resources and ultimately impeding athletic performance (Kahneman, 1973).

In summary, athletes’ access to mobile phones can be increased through environmental factors, which can interfere with normal physiological and psychological states, causing poor interpersonal relationships, triggering interpersonal conflicts, and so on. Furthermore, the likelihood of athletes suffering from MPD can raise due to poor social environments, thus leading them to satisfy the needs through the virtual world and making it difficult for limited psychological resources to be used effectively for training activities. Studies have indicated that athletes who interacted with social media for more than 30 min before training experience a decrease in decision-making performance (*p* = 0.001), and an increase in internal training load (*p* = 0.001) (Fortes et al., 2023). Therefore, coaches and relevant managers should encourage athletes to abstain from social media on smartphones, such as WhatsAPP, Facebook, and Instagram, for at least 2-h before their training sessions, and strictly control and limit the frequency and duration of mobile phone usage by athletes (Fortes et al., 2021). By improving the environmental conditions of training and competition venues, including locker rooms and training grounds, and regularly carrying out psychological counseling, it is effective to guide athletes in developing proper ways of using mobile phones, eliminating external interference, focusing on training and competition, and striving to realize their own value. In addition, the coaching level and quality of coaches also play a vital role in influencing the MPD of athletes. Gearity and Murray (2011) have argued that bad coaches may be at greater risk in a number of ways, such as the suppression of mental skills and distraction of athletes, and thus eventually divide the team. The external factors mentioned above can also exacerbate psychological burden and affect the training state of athletes, further increasing the risk of developing MPD. Therefore, coaches and managers should pay attention to their own behavioral norms and optimize the training environment to eliminate the impacts of potential triggers of MPD and ensure that athletes are in an optimal psychological state.

## 3 Neurobiological mechanisms of the effects of MPD on athletic performance

Research on the neurobiological mechanisms underlying the effects of MPD on athletic performance has focused primarily on the brain reward system (BRS), which involves the areas of brain responsible for reward and loss of impulse control (Liu et al., 2019). The BRS comprises primarily the lower part of the cerebral cortex (e.g., amygdala, hippocampus and ventral striatum) as well as cortical areas responsible for executive functions (e.g., the left prefrontal cortex, orbitofrontal cortex, anterior cingulate cortex and insula) (Morgenstern et al., 2013). The lower part of the cerebral cortex primarily mediates neural processes related to emotion, memory and reward, while the cortical areas responsible for executive functions are involved in cognitive processes such as behavioral regulation and decision-making, which play a significant role in the control and direction of movement. For instance, when athletes with MPD use mobile phones, the brain’s reward pathways may be stimulated due to the stimulation of mobile phone content, while dopamine with neurotransmitters will be released by the ventral tegmental area. These dopamine molecules rapidly bind to dopaminergic receptors in the ventral striatum, enabling athletes with MPD to produce a sustainable sense of excitement and pleasure (Sharma et al., 2021). Moreover, the amygdala, as a key site for emotional processing and memory formation, and the hippocampus, as an important structure for learning and memory, reinforce the association between MPD and pleasurable memory in athletes and thus form the conditioned reward effect for mobile phone usage.

Therefore, athletes with MPD may be addicted to mobile phones for short-term, high-intensity reward satisfaction from mobile phones, prompting the body to secrete large amounts of dopamine. Thus these dopamine molecules can further increase the degree of MPD and weaken athlete’s driving force for delayed reward processes such as training and competition, which will result in reduced vision, attention, reactions, and ultimately cause serious impairment of athletic performance. According to the brain reward theory (Berridge and Robinson, 2016), as behavioral dependence deepens, the function of the cerebral cortex will also be adversely affected, with the decision-making and executive function of athletes being debilitated (Benarroch, 2019; Xiao et al., 2022). Evidence has suggested that the decision-making of athletes relies on the abilities of the central nervous system to process information and motor responses, and that athletes are often required to make quick decisions in multitasking sports scenarios to cope with complex environmental changes (Hughes and Dai, 2023). However, the negative effects of MPD on the central nervous system may lead to an attenuation of information processing and reaction capacity, which could further restrict decision-making, distraction and athletic performance (Encel et al., 2017; Fortes et al., 2019).

Neuroimaging studies of social behaviors have demonstrated that social media use recruits brain network regions, including the prefrontal cortex (PFC), dorsomedial PFC (DMPFC), ventromedial PFC (VMPFC), bilateral temporoparietal junction (TPJ), anterior temporal lobes (ATL), inferior frontal gyri (IFG), and posterior cingulate cortex/precuneus (PCC) (Wolf et al., 2010; Schurz et al., 2014). Among these, the PFC and VMPFC are responsible for attention, processing information, cognitive interference control and decision-making during physical effort (Franco-Alvarenga et al., 2019; Friehs et al., 2020). Prolonged problematic use of mobile phones may lead to physical and mental fatigue and impair internal training load, heart rate variability and cognitive interference control, thereby adversely affecting athletic performance (Fortes et al., 2022a). Finally, mobile phones also emit microwaves and high electromagnetic modulated radiation, which interferes with synaptic neurotransmitters (dopamine, endogenous opioids, etc.) in the BRS, resulting in disruptions to the functions of the nervous system, endocrine system and immune system, as well as to some unique structures of the organism, including the brain waves, the blood–brain barrier and the pineal gland (De La Puente et al., 2007; Sharma et al., 2021). Studies have demonstrated that behavioral dependence involves multiple neurotransmitter systems, among which dopamine seems to be the most dynamic because it intervenes to a greater or lesser measure on a neuronal reward circuit known as the mesolimbic dopaminergic system (De La Puente et al., 2007). The radiation from mobile phones may interfere with the dopaminergic neural circuits in the BRS by affecting the dopaminergic nerve conduction in the synaptic cleft. Therefore, the attention, emotion regulation and decision-making abilities of athletes may be impaired by this interference to some extent. In summary, MPD, which mainly affects the reward pathway in the BRS and emits microwave radiation, could cause dysfunction of brain regions in athletes, potentially restricting athletic performance.

## 4 Relevant theoretical models of effects of MPD on athletic performance

### 4.1 The social cognitive model of self-regulation

**Figure 1:**
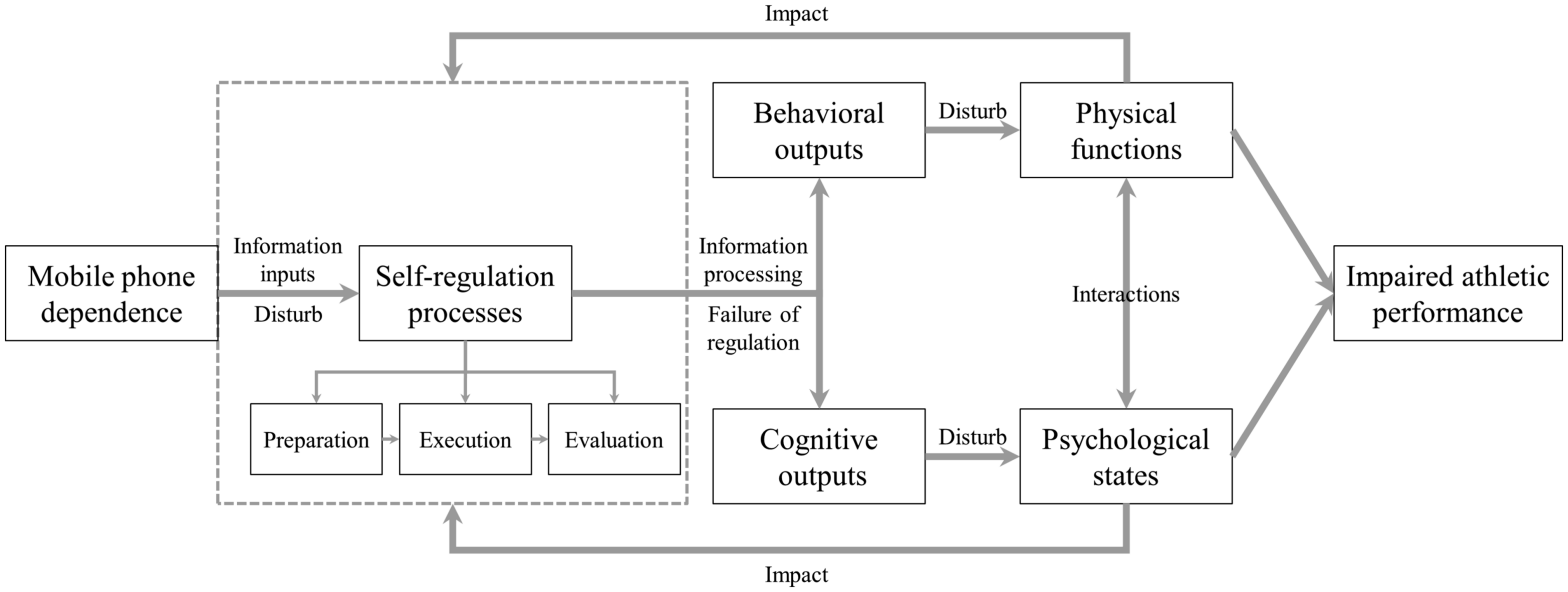
Social cognitive model of self-regulation.

The social cognitive model of self-regulation suggested that self-regulation of athletes can be seen as a process of interaction between athletes (including behavior and cognition) and the environment (Figure 1). Self-regulation refers to self-generated thoughts, feelings, and actions that are planned and cyclically adapted to the attainment of personal goals (Zimmerman, 2000). The process of self-regulation involves three phases: preparation, execution (or volitional control), and evaluation, each of which also includes some key attributes required for athletes to successfully master and execute sports skills, such as self-efficacy, autonomous motivation, goal setting, and attentional capacity (Zimmerman, 2000). Among them, the preparation phase is the process by which the training or competition goals, plans, and strategies are set by the athletes; the execution phase involves the behavior of athletes that occurs during the implementation of an activity; and the evaluation phase refers to the self-observation and judgement of athletes at the end of activity (McCormick et al., 2019). The model pointed out that athletes may suffer from an imbalance in self-regulation due to the long-term effects of MPD, resulting in impairment of the preparation, execution, and evaluation phases, and further outputting disturbing signals to the behavior and cognition of athletes. Physical functions (physical capability, eyesight, sleep quality, etc) and psychological states (mood, engagement, resilience, etc.) are under the constant influence of these signals, with athletic performance being impaired. This finding is consistent with the self-regulation theory proposed by Köpetz et al. (2013), suggesting that MPD stems primarily from a failure of self-regulation, and that growing desires cannot be controlled due to the lack of self-regulation, making it difficult for athletes to eliminate MPD.

Studies have demonstrated that when frequently using mobile phones during training or competition, athletes will focus on irrelevant cues or outcomes (preparation phase), and such athletes cannot be able to fully engage in the execution of sports skills due to the adverse effects of this behavior such as distraction and a lack of self-preparation (execution phase), and the negative comments issued on social media will be checked by them (evaluation phase). Finally, it is easy for these athletes to make misattributions and non-adaptive judgments, leading to an imbalance in self-regulation (Durand-Bush and DesClouds, 2018). In a controlled trial examining the relationship between self-regulation and athletic performance in a 10-kilometre cycling time trial, it was found that athletes with suppressed levels of self-regulation had lower maximum heart rate, mean power outputs, and greater physical exertion when completing the race compared with normal athletes (Wagstaff, 2014). This suggests that the self-regulation disorder of athletes not only adversely affect their physiological function, but also limit athletic performance. In contrast, an intervention trial conducted on self-regulation strategies for college athletes has shown that after self-regulation strategies were applied to college athletes, such as reflection, time management, and cognitive restructuring, the stress levels of these athletes decreased significantly at different time periods, including pre to mid-intervention, post-intervention, and end-of-intervention, which contributed to athletic performance (Dubuc-Charbonneau and Durand-Bush, 2015). In summary, based on the perspective of the social cognitive model of self-regulation, MPD can be regarded as the result of a failure of self-regulation in athletes. Athletes with poor self-regulation are more susceptible to adverse influence from mobile phones, leading to self-regulation disorders and imbalance between the individual and the environment, which ultimately leads to a decline in athletic performance. In light of the negative influence of MPD on self-regulation in athletes, the self-regulation training, including mindfulness and attention control training, should be implemented by coaches and sports administrators, in a bid to help athletes resist negative external influence, and maintain a relative balance between athletes and their surroundings.

### 4.2 The integrative model of self-control

**Figure 2:**
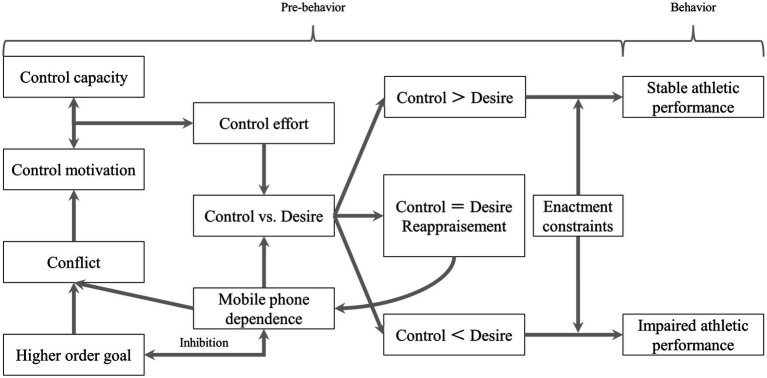
Integrative model of self-control.

Self-control refers to the ability to maintain long-term goals by resisting inner desires and external temptations (Tangney et al., 2018). Athletes with weak self-control are less able to resist automated habitual actions, such as MPD, and tend to pursue immediate enjoyment, current desires, and novel experiences (Zhong et al., 2021). The integrated model of self-control suggested that self-control consists of control capacity, control motivation, control effort, high order goal (better athletic performance), behavioral dependence (MPD), conflict, and enactment constraints (Figure 2) (Kotabe and Hofmann, 2015). Among them, the conflict will be created by the high order goal and MPD, by which the control capacity and control motivation work together to determine the degree of control effort to counteract the desire caused by MPD (Kotabe and Hofmann, 2015). The enactment constraints refer to the environmental factors that limit the behavior of athletes, such as coaches and training conditions. For athletes with normal mobile phone usage, whose degree of the control effort is greater than the desire for MPD, thus there is no behavioral dependence and the stable athletic performance is achieved. For athletes with MPD, however, desires override the control effort, which will lead to the failure of self-control, triggering a range of negative effects of MPD, including impaired athletic performance. Based on the perspective of this model, the control efforts of athletes can be seriously depleted by behavioral dependencies such as MPD. Faced with the pressure generated by training or competition, athletes with weak self-control are less able to suppress instant entertainment and social rewards (the desire to use mobile phones), making it difficult for them to perform at a normal level of athletic performance.

Self-control in athletes was associated with autonomous motivation (Jordalen et al., 2016, 2018). Athletes with higher levels of autonomous motivation and self-control tended to act in accordance with their values and needs, control their own behaviors in a more flexible manner, resist temptations (Deci and Ryan, 2013), and persist with training activities and long-term goals, in order to achieve delayed satisfaction with outstanding athletic performance (Tangney et al., 2018). For athletes with MPD, however, the failure of self-control makes it difficult to make rational judgments and maintain focus in training and competition, resulting in an inability to achieve optimal athletic performance. In contrast, the risk-buffering hypothesis states that self-control can be regarded as a protective mechanism against MPD, and athletes with greater self-control are able to effectively resist the temptation of mobile phones (Siegmann et al., 2018). For such athletes, the time spent interacting with mobile phones can be reasonably controlled, with more control resources applied to the execution of training and competition tasks, thus improving task focus and maintaining the stability of athletic performance (Niu et al., 2020; Zhong et al., 2021). Moreover, by following preset plans, athletes with greater self-control are able to concentrate on improving performance and attaining goals in intense sports, with athletic performance being enhanced in stressful environments (Englert, 2017). In summary, based on the integrative model of self-control, self-control can be seen as the key to moderating the effects of MPD on athletic performance. Sufficient control resources and motivations should be developed and allocated to various training tasks in a rational manner by athletes, so as to avoid crossing the “safety line” of mobile phone usage, to ensure that athletes are protected from the negative effects of excessive mobile phone usage, and to achieve optimal athletic performance.

## 5 Discussion

With the popularity of mobile electronic devices, people increasingly rely on mobile phones for information acquisition, social communication and entertainment in daily life. However, the likelihood of developing MPD will be increased by chronic problematic use of mobile phones. For athletes, due to long-term focus on training and competition, while facing greater pressure, such group are more inclined to regard mobile phones as “stress release device” and “psychological refuge”. This implies that athletes are more susceptible to the detrimental effects of MPD, including distraction, mental fatigue, impaired decision-making, sleep disturbance, and poor social relationship, which can seriously hinder athletic performance (Stothart et al., 2015; Thun et al., 2015; Isoard-Gautheur et al., 2016; Romyn et al., 2016; Wachsmuth et al., 2018; Charest and Grandner, 2020; DesClouds and Durand-Bush, 2021; Fortes et al., 2021, 2022a,b, 2023; Alix-Fages et al., 2023; Lima-Junior et al., 2024). Therefore, early detection and effective intervention of MPD problems in athletes have become necessary and significant for improving their training and competition performance.

Based on individual factors, environmental factors, and relevant theoretical models, the present study examined the impacts of MPD on athletic performance, and explained the underlying mechanisms from a neurobiological perspective and relevant theories. First of all, grounded in the general stress theory, self-determination theory, compensatory internet use theory, and uses and gratifications theory, the restrictive effects of MPD on athletic performance through different pathways have been extensively explored. Furthermore, the mechanisms underlying the effects of MPD on athletic performance have been elucidated from the neurobiological perspective, and it has been suggested that the BRS may play a dominant and decisive role in activating the impacts of MPD on athletic performance. Finally, various models, notably the social-cognitive model of self-regulation and the integrated model of self-control, have been used to explain the effects of MPD on athletic performance. At present, although many valuable results have been obtained in this regard, there are still some problems that need to be addressed in future studies.

The effects of MPD on athletic performance and potential mediating and moderating variables that may exist within the process should be further explored and examined. The process of MPD affecting athletic performance is bound to be influenced by many mediating variables. Existing studies mostly focus on the individual level of athletes, including attentional resources (DesClouds and Durand-Bush, 2021), mental fatigue (Fortes et al., 2022a), and inhibitory control (Lima-Junior et al., 2024). While little consideration is given to the unique sociocultural factors of the sports environment, such as training atmosphere, coach style, and teammate relationships, which may facilitate or inhibit the impacts of MPD on athletic performance through moderating effects. Moreover, some positive psychological factors may have a protective role in the effects of MPD on athletic performance, including psychological resilience, coaching support, and sense of self-worth. For instance, athletes who have more positive evaluations of their own worth and ability may not be at risk of developing MPD, let alone gaining a sense of accomplishment from MPD. Instead, such athletes are more inclined to realize their self-worth through the pursuit of excellence, which helps reduce the likelihood of MPD. Therefore, it is necessary for future research to broaden the perspective, and take the relevant sociocultural and protective factors into consideration. And the potential limiting or protective roles of these factors in this process should be further examined, in order to interpret the relationship between MPD and athletic performance more comprehensively and accurately.

The relationships of MPD dynamically affecting athletic performance through a vertical research perspective should be tracked. It is difficult to establish causal relationships between variables because the impacts of MPD on athletic performance from a vertical research perspective have rarely been examined by previous studies. As sports training is a chronic and dynamic process, athletes will experience many changes in terms of physical functions and psychological states during different periods, and the impacts of MPD on athletic performance vary considerably. In light of few vertical research results, vertical samples of athletes at different training periods and stages should be constructed to monitor the fluctuation relationship between MPD and athletic performance. Moreover, the evolution process, typical symptoms, development patterns, and harm of MPD in athletes at different training stages should be examined, explored and analyzed based on the qualitative research results. This will provide effective treatment programs for psychological counseling and experimental interventions targeted and requisite for athletes.

Based on brain reward mechanisms, research on the neurobiological mechanisms by which MPD affects athletic performance should be further expanded. At present, studies on the neurobiological mechanisms between the two mainly have focused mainly on brain regions linked to reward-activated pathways and the effects of microwave radiation on brain structures (De La Puente et al., 2007; Liu et al., 2019; Sharma et al., 2021), but the specific mechanisms by which MPD affects the structure and function of brain regions still need to be further refined. Evidence has indicated that MPD is similar to other types of behavioral dependence such as gambling, smoking, and shopping, that is, they are all associated with structural brain abnormalities (Xiao et al., 2022). In light of the current understanding of the neurobiological mechanisms underlying the effects of MPD on athletic performance, the following interventions could be considered in future research: (1) The cognitive-behavioral interventions could be adopted to help athletes change their thinking patterns and behaviors, and personalize strategies to resist excessive mobile phone usage; (2) The biofeedback training could be employed to strengthen the self-control and attention management abilities of athletes, including eye movement techniques, brain waves, heart rate variability and other indicators, allowing athletes to establish the correct pattern of brain activity; (3) Based on functional magnetic resonance imaging, the relevant changes in brain reward regions of athletes during activation could be identified to monitor the modulation of the dopamine conduction system; and (4) The volume and intensity of training should be scientifically regulated, and high-intensity interval training could be applied to promote the release of endorphins in the brain, thus replacing the rapid reward pathway caused by MPD.

Based on empirical and intervention research combined with interdisciplinary perspectives, the theoretical model and framework of the impacts of MPD on athletic performance should be validated, developed and enriched. The restrictive effects of MPD on athletic performance have been explained by the social cognitive model of self-regulation and the integrative model of self-control from different disciplinary perspectives (Zimmerman, 2000; Kotabe and Hofmann, 2015). However, the factors involved in these models are relatively simple, focusing only on self-regulation and self-control at the individual level, and ignoring the influence of external factors in the social environment. Therefore, the scientificity and effectiveness of existing theoretical models should be examined by designing more rigorous randomized controlled trials and intervention programs, on the basis of which these models can be further developed and their predictive and explanatory credibility improved. In addition, the existing models are mainly derived from the filed of psychology, which suggests that the relevant theories and methods regarding neuroscience, cognitive science, social science and other fields should be absorbed, constructing an interdisciplinary and comprehensive model combined with the results of intervention experiments, with a view to provide theoretical support and guidance for improving the symptoms of MPD in athletes, returning to normal life, and restoring a healthy state of mind and body.

## Author contributions

ZM: Writing – review & editing, Writing – original draft, Visualization, Validation, Resources, Methodology, Investigation, Formal analysis, Conceptualization. YZ: Writing – original draft, Visualization, Investigation, Conceptualization. QF: Writing – review & editing, Visualization, Resources, Conceptualization. SUL: Writing – review & editing, Visualization, Methodology, Conceptualization. SIL: Writing – review & editing, Validation, Supervision, Resources, Project administration, Methodology, Investigation, Funding acquisition, Formal analysis, Conceptualization.

## References

[ref1] Agnew R. (1992). Foundation for a general strain theory of crime and delinquency. Criminology 30, 47–88. doi: 10.1111/j.1745-9125.1992.tb01093.x

[ref2] Alix-Fages C. Baz-Valle E. González-Cano H. Jiménez-Martínez P. Balsalobre-Fernández C. (2023). Mental fatigue from smartphone use or Stroop task does not affect bench press force–velocity profile, one-repetition maximum, or vertical jump performance. Mot. Control. 27, 631–644. doi: 10.1123/mc.2022-0133 37024109

[ref3] Ayyildiz E. Besler H. K. (2022). Examination of social media addiction and sleep behavior of athletes: a study on athletes in universities. J. Educ. Iss. 8, 124–134. doi: 10.5296/jei.v8i3.20240

[ref4] Bangsbo J. (2015). Performance in sports–with specific emphasis on the effect of intensified training. Scand. J. Med. Sci. Sports 25, 88–99. doi: 10.1111/sms.12605, PMID: 26589122

[ref5] Bano N. Khan M. A. Asif U. de Beer J. Rawass H. (2021). Effects of nomophobia on anxiety, stress and depression among Saudi medical students in Jeddah, Saudi Arabia. J. Pak. Med. Assoc. 71, 1–11. doi: 10.47391/JPMA.983, PMID: 34057935

[ref6] Benarroch E. E. (2019). Insular cortex: functional complexity and clinical correlations. Neurology 93, 932–938. doi: 10.1212/WNL.0000000000008525, PMID: 31645470

[ref7] Berridge K. C. Robinson T. E. (2016). Liking, wanting, and the incentive-sensitization theory of addiction. Am. Psychol. 71, 670–679. doi: 10.1037/amp0000059, PMID: 27977239 PMC5171207

[ref8] Billieux J. (2012). Problematic use of the mobile phone: a literature review and a pathways model. Curr. Psychiatr. Rev. 8, 299–307. doi: 10.2174/157340012803520522

[ref9] Billieux J. Maurage P. Lopez-Fernandez O. Kuss D. J. Griffiths M. D. (2015). Can disordered mobile phone use be considered a behavioral addiction? An update on current evidence and a comprehensive model for future research. Curr. Addict. Rep. 2, 156–162. doi: 10.1007/s40429-015-0054-y

[ref10] Brouzos A. Papadopoulou A. Baourda V. C. (2024). Effectiveness of a web-based group intervention for internet addiction in university students. Psychiatry Res. 336, 1–9. doi: 10.1016/j.psychres.2024.115883, PMID: 38598947

[ref11] Charest J. Grandner M. A. (2020). Sleep and athletic performance impacts on physical performance, mental performance, injury risk and recovery, and mental health. Sleep Med. Clin. 15, 41–57. doi: 10.1016/j.jsmc.2019.11.005, PMID: 32005349 PMC9960533

[ref12] David J. L. Powless M. D. Hyman J. E. Purnell D. M. Steinfeldt J. A. Fisher S. (2018). College student athletes and social media: the psychological impacts of twitter use. Int. J. Sport Commun. 11, 163–186. doi: 10.1123/ijsc.2018-0044

[ref13] De La Puente M. P. Balmori A. Garcia P. (2007). Addiction to cell phones. Are there neurophysiological mechanisms involved. Proyecto 61, 1–6,

[ref14] Deci E. L. Ryan R. M. (2013). Intrinsic motivation and self-determination in human behavior. Berlin: Springer Science & Business Media.

[ref15] DesClouds P. Durand-Bush N. (2021). Smartphones and varsity athletes: a complicated relationship. Front. Sports Act. Living 2, 1–14. doi: 10.3389/fspor.2020.560031, PMID: 33490951 PMC7815595

[ref16] Dubuc-Charbonneau N. Durand-Bush N. (2015). Moving to action: the effects of a self-regulation intervention on the stress, burnout, well-being, and self-regulation capacity levels of university student-athletes. J. Clin. Sport Psychol. 9, 173–192. doi: 10.1123/jcsp.2014-0036

[ref17] Durand-Bush N. DesClouds P. (2018). Smartphones: how can mental performance consultants help athletes and coaches leverage their use to generate more benefits than drawbacks? J. Sport Psychol. Action 9, 227–238. doi: 10.1080/21520704.2018.1496211

[ref18] Elhai J. D. Dvorak R. D. Levine J. C. Hall B. J. (2017). Problematic smartphone use: a conceptual overview and systematic review of relations with anxiety and depression psychopathology. J. Affect. Disord. 207, 251–259. doi: 10.1016/j.jad.2016.08.030, PMID: 27736736

[ref19] Encel K. Mesagno C. Brown H. (2017). Facebook use and its relationship with sport anxiety. J. Sports Sci. 35, 756–761. doi: 10.1080/02640414.2016.1186817, PMID: 27214782

[ref20] Englert C. (2017). Ego depletion in sports: highlighting the importance of self-control strength for high-level sport performance. Curr. Opin. Psychol. 16, 1–5. doi: 10.1016/j.copsyc.2017.02.028, PMID: 28813329

[ref21] Fiedler R. Heidari J. Birnkraut T. Kellmann M. (2023). Digital media and mental health in adolescent athletes. Psychol. Sport Exerc. 67, 102421–102410. doi: 10.1016/j.psychsport.2023.102421, PMID: 37665874

[ref22] Fortes L. S. Fonseca F. S. Nakamura F. Y. Barbosa B. T. Gantois P. de Lima-Júnior D.. (2021). Effects of mental fatigue induced by social media use on volleyball decision-making, endurance, and countermovement jump performance. Percept. Mot. Skills 128, 2745–2766. doi: 10.1177/00315125211040596, PMID: 34404292

[ref23] Fortes L. S. Gantois P. de Lima-Junior D. Barbosa B. T. Ferreira M. E. C. Nakamura F. Y.. (2023). Playing videogames or using social media applications on smartphones causes mental fatigue and impairs decision-making performance in amateur boxers. Appl. Neuropsychol. Adult 30, 227–238. doi: 10.1080/23279095.2021.1927036 34061684

[ref24] Fortes L. S. Lima Junior D. Costa Y. P. Albuquerque M. R. Nakamura F. Y. Fonseca F. S. (2022a). Effects of social media on smartphone use before and during velocity-based resistance exercise on cognitive interference control and physiological measures in trained adults. Appl. Neuropsychol. Adult 29, 1188–1197. doi: 10.1080/23279095.2020.1863796, PMID: 33372542

[ref25] Fortes L. S. Lima-Junior D. Nascimento-Júnior J. R. Costa E. C. Matta M. O. Ferreira M. E. (2019). Effect of exposure time to smartphone apps on passing decision-making in male soccer athletes. Psychol. Sport Exerc. 44, 35–41. doi: 10.1016/j.psychsport.2019.05.001

[ref26] Fortes L. S. Nakamura F. Y. Lima-Junior D. Ferreira M. E. Fonseca F. S. (2022b). Does social media use on smartphones influence endurance, power, and swimming performance in high-level swimmers? Res. Q. Exerc. Sport 93, 120–129. doi: 10.1080/02701367.2020.1810848, PMID: 32930640

[ref27] Franco-Alvarenga P. E. Brietzke C. Canestri R. Goethel M. F. Hettinga F. Santos T. M.. (2019). Caffeine improved cycling trial performance in mentally fatigued cyclists, regardless of alterations in prefrontal cortex activation. Physiol. Behav. 204, 41–48. doi: 10.1016/j.physbeh.2019.02.009, PMID: 30742838

[ref28] Friehs M. A. Klaus J. Singh T. Frings C. Hartwigsen G. (2020). Perturbation of the right prefrontal cortex disrupts interference control. NeuroImage 222:117279. doi: 10.1016/j.neuroimage.2020.117279, PMID: 32828926

[ref29] Gantois P. Caputo Ferreira M. E. de Lima-Junior D. Nakamura F. Y. Batista G. R. Fonseca F. S.. (2020). Effects of mental fatigue on passing decision-making performance in professional soccer athletes. Eur. J. Sport Sci. 20, 534–543. doi: 10.1080/17461391.2019.1656781, PMID: 31424354

[ref30] Gearity B. T. Murray M. A. (2011). Athletes’ experiences of the psychological effects of poor coaching. Psychol. Sport Exerc. 12, 213–221. doi: 10.1016/j.psychsport.2010.11.004

[ref31] Gocer I. Oniz M. (2023). The effect of digital addiction on athletic performance. J. Exerc. Sci. Phys. Act. Rev. 1, 1–13. doi: 10.5281/zenodo.8399841

[ref32] Gould D. Nalepa J. Mignano M. (2020). Coaching generation Z athletes. J. Appl. Sport Psychol. 32, 104–120. doi: 10.1080/10413200.2019.1581856

[ref33] Gupta N. (2023). Impact of smartphone overuse on health and well-being: review and recommendations for life-technology balance. J. Appl. Sci. Clin. Pract. 4, 4–12. doi: 10.4103/jascp.jascp_40_22

[ref34] Hamlin M. J. Wilkes D. Elliot C. A. Lizamore C. A. Kathiravel Y. (2019). Monitoring training loads and perceived stress in young elite university athletes. Front. Physiol. 10, 1–12. doi: 10.3389/fphys.2019.00034, PMID: 30761016 PMC6361803

[ref35] Harris B. Regan T. Schueler J. Fields S. A. (2020). Problematic mobile phone and smartphone use scales: a systematic review. Front. Psychol. 11, 1–24. doi: 10.3389/fpsyg.2020.00672, PMID: 32431636 PMC7214716

[ref36] Huang H. Wan X. Lu G. Ding Y. Chen C. (2022). The relationship between alexithymia and mobile phone addiction among mainland chinese students: a meta-analysis. Front. Psych. 13, 1–17. doi: 10.3389/fpsyt.2022.754542, PMID: 35222110 PMC8866180

[ref37] Hughes G. Dai B. (2023). The influence of decision making and divided attention on lower limb biomechanics associated with anterior cruciate ligament injury: a narrative review. Sports Biomech. 22, 30–45. doi: 10.1080/14763141.2021.1898671, PMID: 33821758

[ref38] Isoard-Gautheur S. Trouilloud D. Gustafsson H. Guillet-Descas E. (2016). Associations between the perceived quality of the coach–athlete relationship and athlete burnout: an examination of the mediating role of achievement goals. Psychol. Sport Exerc. 22, 210–217. doi: 10.1016/j.psychsport.2015.08.003

[ref39] Ivarsson A. Johnson U. Andersen M. B. Tranaeus U. Stenling A. Lindwall M. (2017). Psychosocial factors and sport injuries: meta-analyses for prediction and prevention. Sports Med. 47, 353–365. doi: 10.1007/s40279-016-0578-x, PMID: 27406221

[ref40] Jahrami H. Fekih-Romdhane F. Saif Z. Bragazzi N. L. Pandi-Perumal S. R. BaHammam A. S.. (2022). A social media outage was associated with a surge in nomophobia, and the magnitude of change in nomophobia during the outage was associated with baseline insomnia. Clocks Sleep 4, 508–519. doi: 10.3390/clockssleep4040040, PMID: 36278533 PMC9589948

[ref41] Jones M. J. Dawson B. Eastwood P. R. Halson S. L. Miller J. Murray K.. (2021). Influence of electronic devices on sleep and cognitive performance during athlete training camps. J. Strength Cond. Res. 35, 1620–1627. doi: 10.1519/JSC.0000000000002991, PMID: 30741866

[ref42] Jordalen G. Lemyre P.-N. Durand-Bush N. (2016). Exhaustion experiences in junior athletes: the importance of motivation and self-control competencies. Front. Psychol. 7, 1–9. doi: 10.3389/fpsyg.2016.01867, PMID: 27933031 PMC5121120

[ref43] Jordalen G. Lemyre P.-N. Solstad B. E. Ivarsson A. (2018). The role of self-control and motivation on exhaustion in youth athletes: a longitudinal perspective. Front. Psychol. 9:332262. doi: 10.3389/fpsyg.2018.02449, PMID: 30564181 PMC6288308

[ref44] Jowett S. Cockerill I. M. (2003). Olympic medallists’ perspective of the althlete–coach relationship. Psychol. Sport Exerc. 4, 313–331. doi: 10.1016/S1469-0292(02)00011-0

[ref45] Jun S. Choi E. (2015). Academic stress and internet addiction from general strain theory framework. Comput. Human Behav. 49, 282–287. doi: 10.1016/j.chb.2015.03.001

[ref46] Kahneman D. (1973). Attention and effort. Englewood Cliffs: Prentice-Hall.

[ref47] Kardefelt-Winther D. (2014). A conceptual and methodological critique of internet addiction research: towards a model of compensatory internet use. Comput. Human Behav. 31, 351–354. doi: 10.1016/j.chb.2013.10.059

[ref48] Katz E. Blumler J. G. Gurevitch M. (1973). Uses and gratifications research. Public Opin. Q. 37, 509–523. doi: 10.1086/268109

[ref49] Kawyannejad R. Mirzaei M. Valinejadi A. Hemmatpour B. Karimpour H. A. AminiSaman J.. (2019). General health of students of medical sciences and its relation to sleep quality, cell phone overuse, social networks and internet addiction. Biopsychosoc. Med. 13, 12–17. doi: 10.1186/s13030-019-0150-7, PMID: 31114630 PMC6515609

[ref50] Khoshgoftar M. Mazaheri M. A. Tarahi M. J. (2019). The effect of educational intervention based on health belief model to decrease and prevent mobile phone addiction among female high school students in Iran. Int. J. Pediatr.-Mashhad 7, 10175–10185. doi: 10.22038/ijp.2019.40785.3438

[ref51] Köpetz C. E. Lejuez C. W. Wiers R. W. Kruglanski A. W. (2013). Motivation and self-regulation in addiction: a call for convergence. Perspect. Psychol. Sci. 8, 3–24. doi: 10.1177/1745691612457575, PMID: 26069472 PMC4461059

[ref52] Kotabe H. P. Hofmann W. (2015). On integrating the components of self-control. Perspect. Psychol. Sci. 10, 618–638. doi: 10.1177/1745691615593382 26386000

[ref53] Lan Y. Ding J.-E. Li W. Li J. Zhang Y. Liu M.. (2018). A pilot study of a group mindfulness-based cognitive-behavioral intervention for smartphone addiction among university students. J. Behav. Addict. 7, 1171–1176. doi: 10.1556/2006.7.2018.103, PMID: 30418075 PMC6376383

[ref54] Lima-Junior D. Fortes L. S. Ferreira M. E. C. Gantois P. Barbosa B. T. Albuquerque M. R.. (2024). Effects of smartphone use before resistance exercise on inhibitory control, heart rate variability, and countermovement jump. Appl. Neuropsychol. Adult 31, 48–55. doi: 10.1080/23279095.2021.1990927, PMID: 34747667

[ref55] Liu S. Xiao T. Yang L. Loprinzi P. D. (2019). Exercise as an alternative approach for treating smartphone addiction: a systematic review and meta-analysis of random controlled trials. Int. J. Environ. Res. Public Health 16, 1–16. doi: 10.3390/ijerph16203912, PMID: 31618879 PMC6843500

[ref56] Lopez-Fernandez O. (2017). Short version of the smartphone addiction scale adapted to Spanish and French: towards a cross-cultural research in problematic mobile phone use. Addict. Behav. 64, 275–280. doi: 10.1016/j.addbeh.2015.11.013, PMID: 26685805

[ref57] Malinauskas R. Malinauskiene V. (2019). A meta-analysis of psychological interventions for internet/smartphone addiction among adolescents. J. Behav. Addict. 8, 613–624. doi: 10.1556/2006.8.2019.72, PMID: 31891316 PMC7044583

[ref58] McCormick A. Meijen C. Anstiss P. A. Jones H. S. (2019). Self-regulation in endurance sports: theory, research, and practice. Int. Rev. Sport Exerc. Psychol. 12, 235–264. doi: 10.1080/1750984X.2018.1469161

[ref59] Mehmood A. Bu T. Zhao E. Zelenina V. Alexander N. Wang W.. (2021). Exploration of psychological mechanism of smartphone addiction among international students of China by selecting the framework of the I-PACE model. Front. Psychol. 12, 1–10. doi: 10.3389/fpsyg.2021.758610, PMID: 34867657 PMC8632695

[ref60] Morgenstern J. Naqvi N. H. Debellis R. Breiter H. C. (2013). The contributions of cognitive neuroscience and neuroimaging to understanding mechanisms of behavior change in addiction. Psychol. Addict. Behav. 27, 336–350. doi: 10.1037/a0032435, PMID: 23586452 PMC3700582

[ref61] Niu G. Yao L. Wu L. Tian Y. Xu L. Sun X. (2020). Parental phubbing and adolescent problematic mobile phone use: the role of parent-child relationship and self-control. Child Youth Serv. Rev. 116:105247. doi: 10.1016/j.childyouth.2020.105247

[ref62] Ong N. C. Kee Y. H. Pillai J. S. Lim H. B. Lin Y. C. (2022). Problematic mobile phone use among youth athletes: a qualitative investigation using focus groups. Int. J. Sport Exerc. Psychol. 21, 1–22. doi: 10.1080/1612197X.2022.2152855

[ref63] Pallesen S. Gundersen H. S. Kristoffersen M. Bjorvatn B. Thun E. Harris A. (2017). The effects of sleep deprivation on soccer skills. Percept. Mot. Skills 124, 812–829. doi: 10.1177/0031512517707412, PMID: 28485189

[ref64] Park C. S. (2019). Examination of smartphone dependence: functionally and existentially dependent behavior on the smartphone. Comput. Human Behav. 93, 123–128. doi: 10.1016/j.chb.2018.12.022

[ref65] Romyn G. Robey E. Dimmock J. A. Halson S. L. Peeling P. (2016). Sleep, anxiety and electronic device use by athletes in the training and competition environments. Eur. J. Sport Sci. 16, 301–308. doi: 10.1080/17461391.2015.1023221, PMID: 25790844

[ref66] Sahu M. Gandhi S. Sharma M. K. (2019). Mobile phone addiction among children and adolescents: a systematic review. J. Addict. Nurs. 30, 261–268. doi: 10.1097/JAN.0000000000000309 31800517

[ref67] Schlenker B. R. Leary M. R. (1982). Social anxiety and self-presentation: a conceptualization model. Psychol. Bull. 92, 641–669. doi: 10.1037/0033-2909.92.3.641, PMID: 7156261

[ref68] Schurz M. Radua J. Aichhorn M. Richlan F. Perner J. (2014). Fractionating theory of mind: a meta-analysis of functional brain imaging studies. Neurosci. Biobehav. Rev. 42, 9–34. doi: 10.1016/j.neubiorev.2014.01.009, PMID: 24486722

[ref69] Sharma B. Kumar P. Sharma P. (2021). Smartphone is it “behaviour addiction or substance abuse disorder”: a review to find chemistry behind. Int. J. Pharm. Sci. Res. 12, 1000–1008. doi: 10.13040/IJPSR.0975-8232.12(1).1000-08

[ref70] Siegmann P. Teismann T. Fritsch N. Forkmann T. Glaesmer H. Zhang X. C.. (2018). Resilience to suicide ideation: a cross-cultural test of the buffering hypothesis. Clin. Psychol. Psychother. 25, e1–e9. doi: 10.1002/cpp.2118, PMID: 28853242

[ref71] Skein M. Duffield R. Edge J. Short M. J. Mündel T. (2011). Intermittent-sprint performance and muscle glycogen after 30 h of sleep deprivation. Med. Sci. Sports Exerc. 43, 1301–1311. doi: 10.1249/MSS.0b013e31820abc5a, PMID: 21200339

[ref72] Smith M. R. Zeuwts L. Lenoir M. Hens N. De Jong L. M. Coutts A. J. (2016). Mental fatigue impairs soccer-specific decision-making skill. J. Sports Sci. 34, 1297–1304. doi: 10.1080/02640414.2016.1156241, PMID: 26949830

[ref73] Song A. Song G. Wang H. Niu Q. Yin G. Chen H.. (2023). Prevalence of mobile phone addiction among medical students: a systematic review. Am. J. Transl. Res. 15, 2985–2998, PMID: 37303637 PMC10250977

[ref74] Staiano W. Bonet L. R. S. Romagnoli M. Ring C. (2024). Mental fatigue impairs repeated sprint and jump performance in team sport athletes. J. Sci. Med. Sport 27, 105–112. doi: 10.1016/j.jsams.2023.10.016, PMID: 37957039

[ref75] Stothart C. Mitchum A. Yehnert C. (2015). The attentional cost of receiving a cell phone notification. J. Exp. Psychol. Hum. Percept. Perform. 41, 893–897. doi: 10.1037/xhp0000100, PMID: 26121498

[ref76] Tangney J. P. Boone A. L. Baumeister R. F. (2018). High self-control predicts good adjustment, less pathology, better grades, and interpersonal success. J. Pers. 72, 271–324. doi: 10.4324/9781315175775-5 15016066

[ref77] Thun E. Bjorvatn B. Flo E. Harris A. Pallesen S. (2015). Sleep, circadian rhythms, and athletic performance. Sleep Med. Rev. 23, 1–9. doi: 10.1016/j.smrv.2014.11.003 25645125

[ref78] Trecroci A. Boccolini G. Duca M. Formenti D. Alberti G. (2020). Mental fatigue impairs physical activity, technical and decision-making performance during small-sided games. PLoS One 15, e0238461–e0238412. doi: 10.1371/journal.pone.0238461, PMID: 32903263 PMC7480836

[ref79] Van Cutsem J. Marcora S. De Pauw K. Bailey S. Meeusen R. Roelands B. (2017). The effects of mental fatigue on physical performance: a systematic review. Sports Med. 47, 1569–1588. doi: 10.1007/s40279-016-0672-0 28044281

[ref80] Wachsmuth S. Jowett S. Harwood C. G. (2018). On understanding the nature of interpersonal conflict between coaches and athletes. J. Sports Sci. 36, 1955–1962. doi: 10.1080/02640414.2018.1428882, PMID: 29343176

[ref81] Wagstaff C. R. (2014). Emotion regulation and sport performance. J. Sport Exerc. Psychol. 36, 401–412. doi: 10.1123/jsep.2013-0257 25226609

[ref82] Ward A. F. Duke K. Gneezy A. Bos M. W. (2017). Brain drain: the mere presence of one’s own smartphone reduces available cognitive capacity. J. Assoc. Consum. Res. 2, 140–154. doi: 10.1086/691462

[ref83] Watson A. Brickson S. (2018). Impaired sleep mediates the negative effects of training load on subjective well-being in female youth athletes. Sports Health 10, 244–249. doi: 10.1177/1941738118757422, PMID: 29420135 PMC5958455

[ref84] Wolf I. Dziobek I. Heekeren H. R. (2010). Neural correlates of social cognition in naturalistic settings: a model-free analysis approach. NeuroImage 49, 894–904. doi: 10.1016/j.neuroimage.2009.08.060, PMID: 19733672

[ref85] Xiao W. Wu J. Yip J. Shi Q. Peng L. Lei Q. E.. (2022). The relationship between physical activity and Mobile phone addiction among adolescents and young adults: systematic review and Meta-analysis of observational studies. JMIR Public Health Surveill. 8, e41606–e41617. doi: 10.2196/41606, PMID: 36515994 PMC9798266

[ref86] Yu S. Sussman S. (2020). Does smartphone addiction fall on a continuum of addictive behaviors? Int. J. Environ. Res. Public Health 17, 1–21. doi: 10.3390/ijerph17020422, PMID: 31936316 PMC7014405

[ref87] Zhong Y. Ma H. Liang Y.-F. Liao C.-J. Zhang C.-C. Jiang W.-J. (2022). Prevalence of smartphone addiction among Asian medical students: a meta-analysis of multinational observational studies. Int. J. Soc. Psychiatry 68, 1171–1183. doi: 10.1177/00207640221089535, PMID: 35422151

[ref88] Zhong W. Wang Y. Zhang G. (2021). The impact of physical activity on college students’ mobile phone dependence: the mediating role of self-control. Int. J. Ment. Health Addict. 19, 2144–2159. doi: 10.1007/s11469-020-00308-x

[ref89] Zimmerman B. J. (2000). Attaining self-regulation: A social cognitive perspective. Amsterdam: Elsevier.

